# The impact of children’s sex composition on parents’ mortality

**DOI:** 10.1186/1471-2458-14-989

**Published:** 2014-09-23

**Authors:** Solveig Glestad Christiansen

**Affiliations:** Department of Economics, University of Oslo, P.O. Box 1095, Blindern, Oslo 0317 Norway

**Keywords:** Mortality, Children’s sex composition, Register data, Norway

## Abstract

**Background:**

This study explores the relationship between children’s sex composition and parents’ mortality in a contemporary western society. It improves on earlier research by using a larger and more representative dataset – constructed from registers and encompassing the entire Norwegian population.

**Methods:**

The analysis is based on discrete-time hazard models, estimated for the years 1980–2008 for women and men born after 1935.

**Results:**

When operationalising sex composition as the “number of boys”, coefficients are insignificant in all specifications. However, when considering the three categories “only boys”, “only girls” and “mixed sex”, I find a small but significant disadvantage of having only girls, compared to having at least one child of each sex, for mothers of two or more children. Having only daughters is associated with a mortality disadvantage compared to having only sons for mothers of two children, but a mortality advantage among mothers with four children. Among women who gave birth to their first child as teenagers, those who have only sons have relatively high mortality. I also find an excess mortality both for mothers of only girls and mothers of only boys in the period 1980–1989.

**Conclusion:**

These results lend some support to the notion that there is a larger benefit of the first son or daughter than the later children of the same sex, and especially in the earliest decade of the study period.

## Background

Many earlier studies have shown a relationship between an individual’s mortality and the number of children he or she has. Generally, the childless have higher mortality than those with children, and those with only one child have higher mortality than those with two. Some authors have also reported an increasing mortality as the number of children exceeds four or five [1–3], while other studies, including one from Norway [4], have shown no such disadvantage at high parities. These relationships have been thought to reflect physiological effects of pregnancies (for women) as well as various types of social effects of having children. For example, children may be a source of emotional satisfaction, and above a certain age they may exert control on parents’ behaviour and provide care and assistance, which may reduce mortality. On the other hand, parenthood may also lead to stress because of economic worries or concerns about the children’s wellbeing. Moreover, there are selective influences: several factors of importance for fertility also affect later health and mortality through other channels. The social effects of parenthood probably vary with the characteristics of the children, such as their personality, education, economic resources, health, and family situation. The children’s sex may also have some importance as a conditioning factor.

Few studies have addressed the effect of children’s sex composition on parents’ mortality, and most of them have considered pre-industrial populations. Using data on Sami women from northern Scandinavia, Helle, Lummaa and Jokela [5] find that having sons increases a woman’s mortality, whereas having daughters has the opposite effect. However, later studies have failed to consistently replicate these results and have usually found identical, or almost identical, effects of sons and daughters on women’s mortality e.g. [6–9]. There has been little research into whether the sex composition of the children affects parents’ mortality in western contemporary societies. A study by Jasienska, Nenko and Jasienski [10] concludes that daughters reduce men’s mortality whereas both sons and daughters increase women’s mortality, and do so to the same extent. However, this investigation includes only 102 women and 163 men born between 1894 and 1937. It also only includes people who had already died at the time of study and had at least one son and one daughter, and excludes those who were single or remarried. Studies based on larger and more representative samples are lacking.

One could argue that there might be diminishing marginal returns to having children of a single sex. For example, even if having one son might benefit the parents and their health, having a second or third son might be less important. Perhaps they would be better off having at least one daughter, who might give them other benefits. In fact, many parents seem to have a preference for mixed-sex offspring. An indication of such sex preferences, is that in the Scandinavian countries there is no effect of the sex of the first born on the probability of having a second child and subsequent fertility is slightly higher among those who have two children of the same sex than those who have one boy and one girl. However, a slightly stronger preference for daughters seems to have developed over the last two decades [11]. This means that Scandinavians still prefer to have at least one child of each sex but now consider it to be more important to have at least one girl than at least one boy.

The purpose of this study is to explore the relationship between children’s sex composition and parents’ mortality in a Nordic setting. The analysis is based on discrete-time hazard models, estimated for the years 1980–2008 for women and men born after 1935 using register data that encompasses the entire Norwegian population. As suggested by earlier studies [10], the effect of a certain sex composition is not necessarily the same for mothers and fathers, so the models are estimated separately for women and men. Furthermore, the effect may vary with certain characteristics of the parents. For example, among those who have support from a spouse, the practical assistance that daughters have often provided may be less crucial. In this study, the conditioning effects of age, education, marital status, age at first birth and period are considered.

Little attention has been devoted to the conceivable underlying mechanisms in the few earlier studies that have addressed the link between children’s sex and parents’ mortality. This paper therefore includes a quite thorough discussion of potential causal effects and selective influences, as well as the variations in these.

Parenthood influences the well-being^a^ and lifestyle as well as economic decisions of the parents. Some of these influences may be contingent on the sex of the child.

A few studies have looked into how the gender composition of children affects lifestyle choices. They have found that having an additional daughter reduces the probability of having an alcohol or drug problem, or smoking [12], and that mothers of first-born daughters weigh less than mothers of first-borns sons. However, fathers whose first-born was a daughter weigh more than a father who’s first-born was a son [13].

It can also be hypothesised that it is more stressful to bring up boys. They are more likely to be hyperactive or diagnosed with attention deficit disorder or autism. More importantly, even boys without any disorders are often seen as more boisterous, noisy and less well behaved than girls. At later ages boys have in recent decades been more likely to drop out of school and be unemployed, which may worry the parents. Moreover, bringing up sons may entail a higher level of economic stress. Studies have found that boys receive more pocket money than girls and that boys consumption is considered more important by parents [14, 15]. A survey by a British bank [16] showed that boys cost on average 23% more to raise than girls.

A US study [17] report that the birth of a son induces a man to increase the number of hours worked more than does the birth of a daughter. Similarly, research employing German data finds that having a first-born son increases fathers’ working hours compared to having a first-born daughter [18]. Moreover, both these studies find that fathers of boys have a higher wage rate than fathers of daughters, so the income advantage of the former is even larger than suggested by difference in working hours. Although fathers of sons work longer hours, there is no evidence that this happens at the expense of time spent with their children. On the contrary, fathers have been found to spend more time with their children – also with their daughters – when they have at least one son, and more time with their sons than with their daughters [19–21]. Even in a gender equal society like Sweden, fathers take out more parental leave following the birth of a son [22].

A much cited study using US data found that having sons reduces the risk of divorce [23]. Later research has, however, failed to consistently replicate this result. For example, a study employing data from 18 countries [24] reveals no difference between one-child couples with a son and one-child couples with a daughter. They do however find that two-child couples with children of the same sex, whether girls or boys, have higher divorce risks than those with one child of each sex, and that sons slightly lower divorce risks in three children families. A Swedish study reports the lowest divorce risk in mixed-sex two-child families, whereas the divorce risk at parity three rises with increasing number of girls [25].

Old people tend to have more social contact with daughters than sons [26, 27] and daughters are more likely than sons to provide care to their elderly parents. However, some studies report that the elderly receive more help from a child of the same sex, and that the reason why women are overrepresented as caregivers is that there are more women than men among the elderly due to men’s higher mortality [28].

All of the effects mentioned above probably have implications for the health of the parents and ultimately their mortality. For example, a higher weight (possibly linked to having sons, for mothers) is a risk factor for several potentially fatal diseases. Working long hours, such as fathers of sons are more inclined to do, may affect health adversely. It has been shown to increase the risk of coronary heart disease and depression [29–31]. Furthermore, men’s possibly stronger involvement with their children if there is a boy in the family may be beneficial for the fathers, as well as for the mothers, in the long run. Even more importantly, divorce – which may also be linked to children’s sex – is strongly associated with mortality (e.g. [32]). Finally, the amount of social support, which daughters are particularly likely to supply to their ageing parents, may affect mortality [33]. Studies based on measurements of subjective health have yet to provide a clear picture. Powdthavee, Wu and Oswald [12] conclude that having an additional daughter induces people to report better subjective health, whereas (in a Middle East setting) Engelman, Agree, Yount and Bishai [34] find a negative association between the number of daughters and reported physical functioning, especially for men.

A few studies have looked at the effect of children’s sex on parents’ well-being. One investigation employing Danish data reports a positive effect on fathers’ well-being if the first-born was a boy [35]. Another study finds that mothers of only sons are the happiest, and that those with a majority of boys display higher levels of happiness than those with other sex compositions of children [36]. Finally, a study reports that fathers of boys are more likely to be happily married [37], which in turn heightens the protective effect of marriage (e.g. [38–40]).

In addition to all of these social effects of having children of a given sex, there are physiological implications for the mother of giving birth to sons. Male foetuses have higher intrauterine growth rates and birth weights and therefore require more maternal energy [41–44]; and women carrying male foetuses experience higher levels of testosterone, which is an immunosuppressant [45, 46].

### Possible variations in the importance of children’s sex

The potential effect of a given sex composition may be conditional on a variety of other factors. I consider these possible conditioning effects by stratifying according to a few characteristics (of the parent): age, period, marital status, education and age at first birth.

The importance of children as caregivers increases with parents’ age, and having at least one daughter may therefore become increasingly advantageous with age. The same is true of the role of a daughter as a source of social contact.

Besides age, marital status may also have an effect on the need for social contact with and help from the children, often daughters, as having a spouse can be a substitute when it comes to practical help and companionship. A wife or husband can also make the partner adopt a healthier lifestyle (e.g. cut down on smoking and drinking) in the same way as children, especially daughters often do.

Education is another possible conditioning factor. The more highly educated have a healthier lifestyle, which means that any pressure concerning life-style changes by children, and especially daughters, will be less important. However, one study [47] found that the highly educated women have a higher preference for girls, which assuming they assess correctly how important a daughter is to them, may mean that having at least one daughter is especially beneficial for this group.

Having a child at an early age is detrimental to health later in life. The effect might be dependent on the sex of the child. Women who become mothers at a very early age, especially teenage mothers, are less likely to be in a relationship with the child’s father or have contact with the father at all. They are also likely to take less education [48]. Having had sons may have been particularly stressful for these women, as sons often require more attention than daughters and are more costly.

Finally, the effect of having a certain sex composition may vary over time. For example, the education of the children has in some studies been shown to have a protective effect on parents’ mortality [49–51], and a few decades ago sons tended to be more educated than daughters. The difference between the sexes in these respects are now much smaller.

## Methods

The study includes all men and women born 1935–1968 who lived in Norway some time between January 1, 1980 and December 31, 2008 and while they had at least one child. The data come from the Norwegian Central Population Register, which includes every person resident in Norway for some time after 1960, each of whom has been assigned a unique identification number (at birth or at the time of immigration). The identification number is used in all kinds of contact with the authorities such as applying for education, paying tax or registering at a new address, thus allowing individual-level data from different registers to be linked. In this study, information on education has been added from the National Education Database operated by Statistics Norway, which includes the highest achieved educational degree based on censuses prior to 1980 and on schools’ reporting thereafter. The available data file reports year of death^b^ (taken from the exact dates in the primary data). The data were used with the permission of Statistics Norway.

In the Norwegian Central Population Register, parents’ identification numbers are included for all children who were born in Norway after 1964, or if born earlier, lived at home according to the 1970 census. Thus, almost complete birth histories can be established for all men and women in the country born after 1935. Unfortunately, given the available data it is not possible to distinguish between biological and adopted children. However the proportion of children who have been adopted is less than 1%.

Discrete-time hazard regression models are estimated, separately for women and men using the Proc Logistic procedure in SAS version 9.3 [52]. I start by considering a model with the number of sons as the independent variable before considering models which compare having at least one child of each sex to those who have only children of one sex.

For each individual, a series of one-year observations was created, starting in January 1980 or in January of the year he or she turned 40 (if born 1940 or later) or immigrated, and ending with the year of death, emigration, or in 2008, whichever came first. Each one-year observation includes an outcome variable, which is whether the person died within that year or not, and several independent variables characterising the situation at the beginning of the year: age, calendar year, educational level, marital status, number of children, and age at first birth. Those who did not have any children at the beginning of the year were excluded. Mathematically the model is **log (1/(1-p)) = bX** where p is the probability of dying, **X** is a vector of covariates and **b** are the estimates. Since death probabilities are low **(1/(1-p)) ≈ p**. An estimate b can therefore be interpreted as p being b times what it is in the reference category.

Among men there were 54 073 deaths during the 12,816,408 person-years of follow-up, while there were 36 325 deaths during the 13,316,760 person-years for women.

Five categories of education are distinguished: compulsory education (10 years of schooling according to the current school system); some secondary education (11–12 years); completed secondary education (13 years); some higher education (14–17 years); and master’s degree or higher (18+ years). When it comes to marital status the categories are: married, divorced, widowed and never married. Using Norwegian register data it is unfortunately not possible to distinguish those living in cohabiting relationships. Age at first birth is divided into the following groups: below 19, 20–22, 23–25, 26–29, 30–34 and above 35 years of age.

The educational level is included in the models because it is an important determinant of fertility as well as mortality (e.g. [53, 54]). For the same reasons, calendar year and age are taken into account. Furthermore, age at first birth is controlled for, as giving birth at a very young age is associated with higher mortality [1, 3, 55] and tends to increase or be positively associated with completed fertility. Marital status affects mortality and is closely linked to reproductive behaviour, though without a clear one-way causality. For example, being unmarried obviously reduces fertility, while the number of children, and perhaps even their sex, are likely to have implications for marital status, which is therefore included in some, but not all, models.

Finally, the association between children’s sex and parents’ mortality may vary with the parent’s age, period, age at first birth, marital status and education. I assess this by estimating the model separately for different categories of these variables.

The exposure time and number of deaths in various categories of age, period, education, marital status, number of children and age at first birth are shown in Table 1.

**Table 1:** **Descriptive statistics (CDR- crude death rate)**

	Men			Women		
	Exposure time	Number of deaths	CDR (per 1000)	Exposure time	Number of deaths	CDR (per 1000)
**Education**						
Compulsory education	3,094,623	19,618	6.34	3,970,690	15,929	4.01
Some secondary education	3,226,322	15,376	4.77	4,519,491	12,867	2.85
Completed secondary education	2,771,992	9,490	3.42	1,706,506	2,553	1.50
Some higher education	2,582,601	7,073	2.74	2,763,219	4,494	1.63
Master’s degree or higher	1,140,870	2,516	2.21	356,854	482	1.35
**Marital status**						
Married	9,957,835	34,347	3.45	9,813,708	22,275	2.27
Never married	666,561	2,342	3.51	581,631	1,294	2.22
Widowed	157,845	1,805	11.43	621,147	3,984	6.41
Divorced	2,034,167	15,579	7.66	2,300,274	8,808	3.83
**Number of children**						
1	1,831,685	9,196	5.02	1,820,757	6,312	3.47
2	5,664,702	21,463	3.79	5,828,087	14,430	2.48
3	3,646,831	14,717	4.04	3,853,061	9,825	2.55
4	1,196,454	5,917	4.95	1,295,294	3,909	3.02
5	476,736	2,780	5.83	519,561	1,849	3.56
**Age at first birth**						
Below 19	319,730	1,866	5.84	1,799,311	6,439	3.58
20-22	2,021,660	10,716	5.30	3,927,521	11,868	3.02
23-25	3,418,994	15,451	4.52	3,390,422	8,667	2.56
26-29	3,799,934	14,775	3.89	2,600,871	5,919	2.28
30-34	2,193,136	7,700	3.51	1,163,814	2,561	2.20
Above 35	1,062,954	3,565	3.35	434,821	872	2.01
**Age**						
40-49	6,733,908	13,236	1.97	6,971,375	8,755	1.26
50-59	4,302,732	20,007	4.65	4,426,953	13,699	3.09
60-73	1,779,768	20,830	11.70	1,918,432	13,871	7.23
**Period**						
1980-89	2,059,959	5,608	2.72	2,123,594	3,299	1.55
1990-99	4,575,997	16,628	3.63	4,719,398	10,878	2.30
2000-08	6,180,452	31,837	5.15	6,473,768	22,148	3.42

## Results

In my first model, sex composition is operationalised as the number of boys. Table 2 displays the results of this regression without controlling for marital status, for men and women, respectively. I control for age, year, education, number of children and age at first birth. Neither for women nor for men is the effect of “Number of boys” statistically significant. The other effects are as expected: mortality is lower and fairly stable when the number of children exceeds one, lower among those who have a higher level of education, and lower for those (especially women) who had their first child later.

**Table 2:** **Relationship between number of boys and parental mortality controlling for parental demographics excluding marital status**

	Men	Women
Year	0.98 (0.97-0.98)	0.99 (0.99-0.99)
Age	1.11 (1.11-1.11)	1.09 (1.09-1.10)
**Education**		
Compulsory education	1	1
Some secondary education	0.76 (0.74-0.78)	0.75 (0.73-0.77)
Completed secondary education	0.65 (0.64-0.67)	0.50 (0.48-0.52)
Some higher education	0.53 (0.52-0.55)	0.55 (0.53-0.57)
Master’s degree or higher	0.42 (0.40-0.44)	0.47 (0.43-0.52)
**Number of children**		
1	1	1
2	0.69 (0.68-0.71)	0.69 (0.66-0.71)
3	0.65 (0.63-0.67)	0.61 (0.59-0.64)
4	0.67 (0.65-0.70)	0.60 (0.57-0.63)
5	0.68 (0.64-0.71)	0.59 (0.56-0.63)
**Age at first birth**		
Below 19	1.34 (1.27-1.40)	1.33 (1.29-1.38)
20-22	1.19 (1.16-1.21)	1.10 (1.07-1.14)
23-25	1	1
26-29	0.91 (0.89-0.93)	0.97 (0.94-1.00)
30-34	0.86 (0.84-0.88)	0.94 (0.90-0.99)
Above 35	0.76 (0.73-0.79)	0.80 (0.74-0.86)
**Children’s sex composition**		
Number of boys	1.00 (0.99-1.01)	1.00 (0.99-1.02)

(Odds ratios with 95% confidence intervals).

Table 3 shows the estimates from models where marital status has been included. We see that the estimated coefficient for the variable “Number of boys” is hardly affected. The estimates for marital status are as seen in earlier studies: there is an advantage of being married compared to being single, whereas the never-married and the divorced are at a greater disadvantage than the widowed.

**Table 3:** **Relationship between number of boys and parental mortality controlling for parental demographics including marital status**

	Men	Women
Year	0.97 (0.966-0.97)	0.99 (0.99-0.99)
Age	1.11 (1.11-1.12)	1.09 (1.09-1.10)
**Education**		
Compulsory education	1	1
Some secondary education	0.79 (0.78-0.81)	0.76 (0.75-0.78)
Completed secondary education	0.69 (0.67-0.70)	0.49 (0.47-0.52)
Some higher education	0.57 (0.55-0.58)	0.55 (0.53-0.57)
Master’s degree or higher	0.46 (0.44-0.48)	0.46 (0.42-0.51)
**Marital status**		
Married	1	1
Never married	2.05 (1.95-2.14)	1.48 (1.40- 1.58)
Widowed	1.65 (1.57-1.73)	1.38 (1.33-1.43)
Divorced	2.16 (2.12-2.20)	1.63 (1.59-1.67)
**Number of children**		
1	1	1
2	0.78 (0.76-0.80)	0.75 (0.73-0.78)
3	0.74 (0.72-0.76)	0.68 (0.66-0.71)
4	0.76 (0.73-0.79)	0.67 (0.64-0.70)
5	0.75 (0.71-0.79)	0.66 (0.64-0.70)
**Age at first birth**		
Below 19	1.27 (1.13-1.34)	1.26 (1.22-1.31)
20-22	1.15 (1.12-1.18)	1.08 (1.05-1.11)
23-25	1	1
26-29	0.92 (0.90-0.94)	0.98 (0.95-1.01)
30-34	0.87 (0.85-0.90)	0.96 (0.92-1.01)
Above 35	0.76 (0.73-0.79)	0.82 (0.99-0.88)
**Children’s sex composition**		
Number of boys	0.99 (0.99-1.01)	1.00 (0.99-1.02)

(Odds ratios with 95% confidence intervals).

In my second model, I replace the number of boys variable with an indicator variable denoting whether all the children are of the same sex. As this variable is always unity for one-child families, Tables 4 and 5 show regression results only for those with two or more children. Controlling for age, period, education, marital status, number of children and age at first birth, I find no significant effect of having only sons or only daughters rather than having at least one child of each sex for men (Table 4). For women, however, there is a statistically significant, though small, mortality disadvantage associated with having only daughters (3%). Very similar estimates are obtained when marital status is excluded from the models (not shown).

**Table 4:** **Effect of having children of only one sex compared to having at least one of each sex, for those with two or more children**

	Men	Women
Only boys	1.01 (0.99-1.04)	1.02 (0.99-1.05)
Only girls	1.02 (0.99-1.05)	1.03 (1.00-1.07)

Controlling for age, period, education, marital status, number of children and age at first birth.

(Odds ratios with 95% confidence intervals).

**Table 5:** **Effect of having children of only one sex in the periods 1980-1989, 1990-1999 and 2000-2008, for those with two or more children**

	Men			Women		
	1980-1989	1990-1999	2000-2008	1980-1989	1990-1999	2000-2008
**Only boys**	1.01 (0.93-1.09)	0.99 (0.99-1.06)	1.03 (0.99-1.06)	1.11 (1.00-1.23)	1.00 (0.95-1.06)	1.01 (0.97-1.06)
**Only girls**	0.99 (0.91-1.07)	1.02 (0.99-1.06)	1.03 (0.99-1.06)	1.15 (1.04-1.24)	1.00 (0.94-1.06)	1.03 (0.99-1.08)

Controlling for age, period, education, marital status, number of children and age at first birth. (Odds ratios with 95% confidence intervals).

When stratifying by level of education, marital status or age, I find no significant effects of children’s sex composition, neither when operationalised as number of boys, nor as only boys, only girls and mixed (results not shown). However, estimating the model separately for the years 1980–1989, 1990–1999 and 2000–2008 I find a disadvantage of having only children of a single sex on the mortality of women for the period 1980–1989 (Table 5).

The other statistically significant effect appears among women who gave birth to their first child as teenagers. In this group, I find a disadvantage of having only sons compared to at least one of each sex (odds ratio 1.10; 95% confidence interval (1.021-1.177)).

Using “having only sons” as the reference category instead of “having at least one of each sex”, in order to compare having only sons to having only daughters (and including parents with only one child) I find that mothers of two daughters have a significantly higher mortality than mothers of two sons (odds ratio 1.05; 95% confidence interval (1.00-1.10)). I also find that mothers of four daughters have a significantly lower mortality than mothers of four sons (odds ratio 0.82; 95% confidence interval (0.69-0.98)) (not shown in tables).

## Discussion

There is almost no knowledge available from contemporary western countries about whether the sex composition – in line with the preferences or not – affects the parents’ mortality. Given the lack of earlier research, a study of the association between children’s sex composition and parents’ mortality based on high-quality data covering an entire national population should be a valuable contribution to the literature. Interestingly, when models are estimated for the period 1980–2008 and using the whole sample, there is only one significant effect: mothers of two or more children who have only girls have a slightly higher (odds ratio 1.03; 95% confidence interval (1.00-1.07)) mortality than those who have at least one child of each sex. These results indicate (weakly) that there is a special value associated with sons. In other words, the benefits supposed to be derived from having a daughter seem to be somewhat smaller than the positive effects of having a son, such as stronger involvement by the father, which probably also benefits the mother. Such a marginally higher value of having a son also accords with the results from some studies of parents’ subjective well-being.

Having only daughters compared to only sons is associated with a mortality disadvantage for mothers with two children, but an advantage for those with four children. The first result again points to a special importance of having sons. The second effect might reflect physiological effects of carrying many male foetuses or that having many sons is in some way burdensome.

According to period-specific models, the high mortality among mothers with only girls, compared to mothers with at least one child of each sex, was confined to the 1980s (odds ratio 1.15; 95% confidence interval (1.04-1.24)), when there was also a significant, but weaker, adverse effect of having only boys (odds ratio 1.11; 95% confidence interval (1.00-1.23)). This finding may reflect that sons and daughters at that time had more distinct roles, with sons for example being able to offer better advice as a result of having more education on average, while daughters to a larger extent contributed as caregivers, and as a consequence it was more important to have at least one child of each sex.

The most surprising result is perhaps that the patterns differ so much between women and men. Women might need children more because they are more often widowed, however an analysis stratified by marital status did not give support to this interpretation.

One might expect the relationship between children’s sex composition and parents’ mortality to vary with age and education, but no such pattern appeared. Only a significant conditioning effect of age at first birth was seen: among women who entered motherhood early, those with only sons had a particularly high mortality (odds ratio 1.10; 95% confidence interval (1.021-1.177)). The main reason might be that it is more stressful for young girls to raise boys as they are often more energetic and boisterous and are a greater drain on the mother, especially if she lacks the support of a partner, as many teenage mothers do.

### Limitations

One limitation of the study is the somewhat limited age range of parents (40–73) that could be included. It may be that some characteristics typically associated with a child’s sex are appreciated by parents primarily at higher ages. An example may be willingness, especially of daughters, to offer social support. The relatively narrow age range also means that the distribution of deaths by cause in the sample differs from the distribution for the population as a whole. It may be that children’s sex is more important for causes of death that are more prevalent at higher ages^c^. Furthermore, given the data it is not possible to determine the possible mechanisms through which children’s sex composition may affect mortality.

Another major limitation of this study is that factors such as previous health status, earlier life crises and socio-economic status other than education could not be controlled for. It is however possible that such factors affect both the children’s sex and the parents’ mortality. Even in settings where sex-specific abortion is very uncommon the male–female sex ratio at birth might not be constant. It has been found to diminish subsequent to disasters or economic downturns (e.g. [56–58]). Furthermore, stressful life events such as having a severely ill partner or child, or bereavement, are associated with a lower sex ratio [59]. Conversely, characteristics indicative of an advantaged situation, economically or otherwise, are associated with high sex ratios: married mothers have been found to give birth to more boys than mothers not living with the father [60, 61], and better educated mothers have more sons [62], as do male billionaires - 60% compared to 51% in the population as a whole [63]. Being married, more highly educated or rich also reduces mortality, while adverse life experiences, such as unemployment or losing a child, has been linked to higher mortality later in life [64]. These factors may therefore in principle give rise to an inverse spurious relationship between the proportion of the children who are sons and parents’ mortality.

## Conclusion

This article finds that there is a small mortality disadvantage among women with two or more children having only daughters, among women who had their first child as a teenager and only have sons, and among mothers in the 1980s who only have children of one sex. Mothers of two daughters and no sons have a higher mortality than mothers of two sons and no daughter. However, mothers of four daughters and no sons have a lower mortality than mothers of four sons and no daughters. The results lend some support to the notion that there is a larger benefit of the first son or daughter than later children of the same sex. This is more pronounced in the earliest decade of the study period, when men and women had more different roles. The findings also indicate (weakly) that supposed benefits of having a daughter (for example because of their special contributions as caregivers), may be somewhat smaller than the positive effects of having a son, such as stronger involvement by the father, which probably also benefits the mother. However, having many sons may be a burden on the mother. In principle, the observed relationships may also reflect selection processes.

Obviously, one cannot conclude from such estimates that men and women who have only girls or boys should try to have an additional child of the opposite sex to improve their health in the long term. Neither do mortality differentials of this modest size serve as a warning that groups of parents with a special sex composition deserve extra attention. Rather, the results should be seen as interesting illustrations of how far-reaching consequences the sex composition of one’s children might have on one’s life.

## Endnotes

^a^A project with the aim of developing measures better reflecting general well-being is being undertaken by Eurostat and the OECD, albeit at a country level. See for example [65, 66].

^b^By law all deaths must be reported to the authorities based on a doctor’s death certificate and will automatically be registered in official statistics.

^c^Research looking at the association between children’s sex and specific causes of death would be an interesting topic for further research. This dataset is also well suited to address other topics such as studying the mortality of those who have lost a child.
